# Unusual neon pink color change in a radiotherapy tattoo

**DOI:** 10.1016/j.jdcr.2026.04.011

**Published:** 2026-04-17

**Authors:** Yana Boroumand, Erika Koh, Ata S. Moshiri, Elizabeth K. Hale

**Affiliations:** Ronald O. Perelman Department of Dermatology, Grossman School of Medicine, New York University, New York, New York

**Keywords:** oncodermatology, pigment change, radiation, tattoo

## Introduction

Medical radiation tattoos are commonly used as guiding markers for treatment and recurrence monitoring.^1^ The tattoos are most commonly black or dark blue in color and are thought to be relatively inert over time.^2^^,^^3^ We report the case of a patient with a radiation tattoo demonstrating an unusual change in color from black to neon pink, a previously unreported phenomenon with multiple possible etiologies. As cancer survivorship increases with advances in cancer therapies, dermatologists must be cognizant of sequelae of cancer therapies, including radiation tattoos and their possible sequelae.

## Case report

**Fig. 1:**
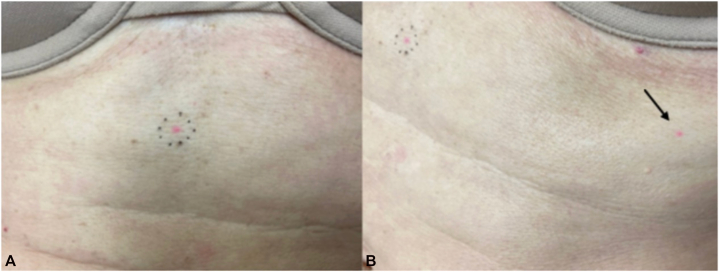
**(A)** The patient’s midline chest demonstrated a neon pink, pinpoint macule on the anterior chest midline between the breasts. **(B)** A similar neon pink, pinpoint macule (*black arrow*) was observed lateral to first macule on the wall of the left side of the chest.

**Fig. 2:**
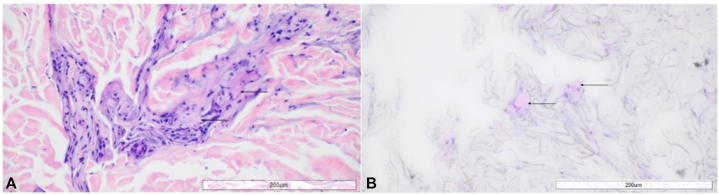
**A,** Stained and **(B)** unstained neon pink amorphous material within histiocytes and between collagen bundles (*black arrows*). (**A,** Hematoxylin-eosin stain; original magnification: **A,** ×400.) (**B,** Original magnification: ×400.)

A 66-year-old woman with history of stage 0 ductal carcinoma in situ of the left breast (treated with lumpectomy followed by 20 fractions of radiotherapy and started on 1 mg anastrozole daily 3 years ago) as well as osteoporosis (on 70 mg alendronate daily for over 10 years) presented to the clinic for an annual total body skin examination. Examination revealed 2 asymptomatic, pinpoint, neon pink macules, including 1 on the anterior aspect of the chest midline between the breasts and 1 directly lateral on the wall of the left side of the chest (Fig 1). Neither lesion had been noted by the patient previously. The patient denied use of coloring agents, such as paints or pens; new topicals; or changes to medications. Four weeks later, she returned for re-examination of the asymptomatic lesions, and the macules were found to be unchanged. A punch biopsy was obtained, demonstrating neon pink-colored deposits within histiocytes and around collagen bundles in the reticular dermis without associated granulomas, spongiosis, inflammatory infiltrate, or fibrosis, suggestive of exogenous tattoo pigment (Fig 2). The dermal ink deposits demonstrated autofluorescence under high intensity light-emitting diode from the fluorescence microscope, suggesting UV reactivity. Of note, the patient previously received a black ink tattoo to the area for radiotherapy treatment of her breast cancer. She denied having received laser treatments to the site. The patient reported obtaining spray tans within the last year and consistent use of sunless tanning lotion for 2 years prior.

## Discussion

The use of medical tattoos for patients planned to receive radiotherapy for cancer treatment is common across cancer centers, and usually requires 3 to 5 tattoos administered using a small needle to deposit permanent ink into the dermis.^2^ As in this case, carbon black ink is the most frequently used tattoo ink.^1^^,^^2^ Although tattoos are known to fade with time, to our knowledge, there are no previous reports in the literature documenting black tattoo color changing to pink. Although less likely, other considerations for tattoo-associated complications in this case included an inflammatory or granulomatous disorder, or allergic reactions.^1^^,^^3^

There are several possibilities as to why the observed color change may have occurred. Tattoo inks are complex formulations containing multiple pigments, a carrier, preservatives, and dispersants to evenly distribute the pigment throughout the solution.^4^ Tattoo ink is thought to change in color over time due to relocation of ink to different skin layers, photochemical decomposition of pigment from UV radiation, as well as phagocytosis and subsequent transportation of pigment to lymph nodes.^5^ Of note, black ink may contain polycyclic aromatic hydrocarbons, which are residual organic molecules from the incomplete combustion during carbon black production that can produce reactive oxygen species in the skin leading to inflammatory reactions.^3^^,^^6^ At any point in these processes, chemical modification and interactions between ink components may have occurred in unexpected ways, altering the tattoo’s appearance. Furthermore, some dark tattoos have a mixture of inks, including black, red, and yellow, and degradation or relocation within the skin of one of the pigments might have caused a perceived change in color of the tattoo.^7^ Although spectroscopy analysis of the ink could not be conducted to confirm this theory, we favor that an unusual combination of inks to form black color hue may have been used in this case leading to the atypical color change. The UV activity seen under fluorescence microscopy further supported the idea that black ink may have been mixed with another color. The color change to the tattoo may also be an unusual late sequelae of cancer treatment, either from a direct effect of ionizing radiation on the ink or from radiation effect on the surrounding tissue leading to fibrosis and decreased vascularity.^8^ However, the patient could not recall a temporal link between receiving radiotherapy and visible changes to the tattoos. Additionally, these stromal changes were not directly seen on biopsy, and this particular color change has not previously been reported. Although visible light can cleave organic pigments in the skin leading to change in appearance of tattoos, the patient denied frequent suntanning. Of note, other inks, including red, green, and pink UV-active ink, have been used in radiation tattoos and should be recognized by dermatologists on skin exam.

An external cause of the change in tattoo appearance is unlikely. Our patient suggested that sunless tanning lotion may have effected this change. However, sunless tanners are not known to penetrate into the reticular dermis where the tattoo pigment is localized and instead utilize dihydroxyacetone sugar to produce brown chromophores within by interacting with proteins in the stratum corneum through the Maillard reaction.^9^ Although other external factors, such as patient use of medication, are unlikely implicated given no known interaction between hormones and pigment deposits in the skin.

As the number of cancer survivors has increased over the past few decades due to significant advancements in cancer therapies, treatment-related dermatologic sequelae, including radiation tattoos, have become more prevalent.^10^ The present case highlights a novel observation of unusual color change within radiation tattoo, raising awareness among clinicians of this unexpected clinical finding.

## Conflicts of interest

None disclosed.
